# PrP^Sc ^detection in formalin-fixed paraffin-embedded tissue by ELISA

**DOI:** 10.1186/1756-0500-4-432

**Published:** 2011-10-21

**Authors:** Eric M Nicholson, Justin J Greenlee, Amir N Hamir

**Affiliations:** 1 Virus and Prion Research Unit, 1920 Dayton Ave., National Animal Disease Center, USDA, Agricultural Research Service, Ames, Iowa 50010, USA; 2 M. D. Anderson Cancer Center, Department of Veterinary Medicine and Surgery-Unit 63, 1515 Holcombe Boulevard, Room TB.4055C, Houston, TX 77030-4009. USA

## Abstract

**Background:**

Formalin-fixed paraffin-embedded tissue is regularly employed in the diagnosis of transmissible spongiform encephalopathies (TSE) by immunohistochemistry (IHC), the standard by which all other TSE diagnostic protocols are judged. While IHC affords advantages over diagnostic approaches that typically utilize fresh or frozen tissue, such as Western blot and ELISA, the process of fixing, staining, and analyzing individual sections by hand does not allow for rapid or high throughput screening. However, preservation of tissues in formalin is not dependent upon the availability of refrigeration.

**Findings:**

Formalin-fixed paraffin-embedded tissues from TSE transmission studies of scrapie in sheep, chronic wasting disease in white-tailed deer or transmissible mink encephalopathy in cattle were cut at 5 μm thickness. Samples containing the tissue equivalent of as little as one 5 μm section can be used to readily discriminate positive from negative samples.

**Conclusions:**

This approach cannot replace IHC but may be used along with IHC as both a more rapid and readily high throughput screen where fresh or frozen tissues are not available or impractical.

## Findings

Due to the lack of a defined immune response or nucleic acid component of the infectious agent, approaches for transmissible spongiform encephalopathy (TSE) diagnosis rely upon methods of immunodetection including immunohistochemistry (IHC), Western blotting and enzyme-linked immunosorbent assay (ELISA)-based approaches for detection of the infectious agent. [1-3] Generally speaking, IHC relies upon formalin fixed paraffin embedded tissues, while Western blotting and ELISA utilize fresh or frozen tissues. Recently, methods have been reported that allow detection of PrP^Sc ^in formalin fixed tissues by Western blot [4-6]. Here we report an extension of this approach to allow ELISA-based detection of PrP^Sc ^in formalin-fixed paraffin-embedded tissues.

### Tissue samples

This study utilized archived paraffin-embedded tissue samples from studies of scrapie in sheep, chronic wasting disease (CWD) in white-tailed deer (WTD) and transmissible mink encephalopathy (TME) in cattle as part of TSE research conducted at the National Animal Disease Center-USDA-ARS (Ames, IA). Animals were cared for and euthanized under National Animal Disease Center approved institutional animal care and use protocols. Samples were collected in 10% neutral buffered formalin prior to standard processing into paraffin blocks, with time in formalin ranging from 7 days to ~450 days. Previous studies of formalin fixed tissues report a marked sensitivity decrease for Western blots on tissues left in formalin for 2 or more years [6]. Based on this observation we limited our analysis to samples with fixation times less than 2 years.

### Sample preparation

The method described here is an extension of previously published methods for Western blotting of formalin-fixed paraffin embedded samples differing only in the method for detecting PrP^Sc ^[4,5]. As previously described, four 5 μm thick tissue sections from each paraffin block were collected into a 1.5 ml centrifuge. To each tube, 150 μl of 0.05 *M *Tris (pH 7.5), 1 *mM *EDTA, and 0.5% Tween 20 was added. The tube was placed at 100°C for 10 min and immediately placed into a dry ice ethanol slurry until frozen. The 10 min boil/freeze cycle was repeated once. The sample was brought to 100°C for an additional 10 min and immediately centrifuged at 3,000 × g for 10 min to separate the paraffin from the aqueous phase while also pelleting the tissue. In the event that the separation of paraffin was incomplete, the tube was reboiled for 10 min and the centrifugation step repeated. The aqueous layer including the tissue pellet was transferred to a clean 1.5 ml tube. At this point, the sample volumes were approximately 120 μl. Tissue disruption by sonication was done in 30 intervals of 40 sec with brief vortex mixing between sonication steps in a bath sonicator filled with ice water.

### Sample Analysis

Following tissue disruption, 100 μL was removed and placed in a clean tube. From this 100 μl sample, a 20 μL sample was used to detect the presence of PrP^Sc ^using the IDEXX HerdChek Bovine Spongiform Encephalopathy-Scrapie Antigen Test Kit ELISA by incorporating the 20 μl sample in place of the 120 μl of tissue homogenate called for in the manufacturer's instructions. The remaining 80 μl was enriched by centrifugation at 186,000 × g for 55 minutes. The supernatant was removed and 20 μl analyzed using the HerdChek kit as described previously. The pellet was resuspended in 20 μl of 0.05 *M *Tris (pH 7.5), 1 *mM *EDTA, and 0.5% Tween 20 and analyzed as described above for both the supernatant and the unenriched sample.

## Results and Discussion

In total, we analyzed 15 samples collected from inoculated animals and 11 samples collected from negative controls (Table 1). PrP^Sc ^was not detected in either the unenriched or enriched samples from any a negative control animal, nor did the supernatant from any animal yield a positive ELISA. We found that 14/15 of the inoculated animals were positive without enrichment and 15/15 after enrichment.

**Table 1:** Detection of PrP^Sc ^in Formalin Fixed Paraffin Embedded Tissues by ELISA.

TSE (species)	Animal #	Supernatant	Unenriched	Enriched
Scrapie (Sheep)	23	0.074	0.76	2.8
	3503	0.11	0.61	2.40
	3515	0.096	0.203	1.36
	3740	0.056	0.19	0.73
	3742	0.061	0.31	1.71
	3506	0.064	0.42	1.27
Neg (Sheep)	0009	0.064	0.063	0.062
	0007	0.083	0.067	0.074
	3527	0.098	0.10	0.13
	0050	0.077	0.059	0.062
	0059	0.074	0.059	0.058
	0004	0.089	0.031	0.089

CWD (WTD)	628	0.083	ND	0.459
	632	0.08	ND	0.322
	648	0.084	ND	0.971
	654	0.086	ND	1.15
Neg (WTD)	645	0.085	ND	0.079
	681	0.075	ND	0.080

TME (Cattle)	520	0.07	0.211	0.508
	521	0.069	0.210	0.618
	522	0.073	**0.162**	0.571
	524	0.060	0.233	1.302
	526	0.065	0.281	0.650
Neg (Cattle)	523	0.074	0.071	0.066
	525	0.074	0.085	0.077
	527	0.072	0.084	0.072

An ELISA cutoff value of 0.18 is used throughout. The only incidence of an inoculated animal below the ELISA cutoff is shown in bold. Neg denotes a negative control animal.

The absence of detectible PrP^Sc ^in the supernatant is consistent with the numeric values of the enriched samples exhibiting a higher signal than the corresponding samples prior to enrichment. Enriched samples were derived from samples 4 times larger than unenriched samples, but signal from enriched samples were not always 4 times greater. Deviations from an actual 4-fold increase in signal as would be expected for the increased concentration were generally small and likely reflect non-linearity of the signal over the detection range or a less than complete recovery in the enrichment centrifugation step or both.

In one instance, an unenriched cattle TME sample was determined to be below the positive cutoff value, however, the enriched sample was clearly positive. Based upon this and the overall enhancement of sensitivity, our recommendations for use of this method include incorporation of the 186,000 × g centrifugation based enrichment.

Of note is that the IDEXX ELISA utilizes a proprietary capture ligand (Seprion ligand) rather than an antibody [7]. This capture ligand binds misfolded PrP^Sc ^protein. Thus, PrP^Sc ^must retain the characteristics of a misfolded protein through the fixation process and the enrichment processing described here or it would not bind the capture surface.

In summary, ELISA based detection of PrP^Sc ^from formalin-fixed paraffin-embedded tissue is a rapid means to detect PrP^Sc ^with the potential for hundreds of sample to be analyzed by an individual in a single day. The detection approach is distinct from IHC and offers the diagnostic and research community an additional tool to detect PrP^Sc^.

## Competing interests

The authors declare that they have no competing interests.

## Authors' contributions

EMN conceived of, designed and conducted the experiments and wrote the manuscript. JJG contributed to manuscript revision and provided tissue samples. ANH provided tissue samples. All authors have read and approved the final manuscript.
